# Features and applications of haplotypes in crop breeding

**DOI:** 10.1038/s42003-021-02782-y

**Published:** 2021-11-04

**Authors:** Javaid Akhter Bhat, Deyue Yu, Abhishek Bohra, Showkat Ahmad Ganie, Rajeev K. Varshney

**Affiliations:** 1 National Center for Soybean Improvement, State Key Laboratory of Crop Genetics and Germplasm Enhancement, Nanjing Agricultural University, Nanjing, China; 2 Crop Improvement Division, ICAR- Indian Institute of Pulses Research (ICAR- IIPR), Kanpur, India; 3 Department of Biotechnology, Visva-Bharati, Santiniketan, 731235 WB India; 4 Center of Excellence in Genomics & Systems Biology, International Crops Research Institute for the Semi-Arid Tropics (ICRISAT), Hyderabad, 502324 India; 5 State Agricultural Biotechnology Centre, Centre for Crop & Food Research Innovation, Food Futures Institute, Murdoch University, Murdoch, WA Australia

**Keywords:** Agricultural genetics, Plant breeding, Plant genetics

## Abstract

Climate change with altered pest-disease dynamics and rising abiotic stresses threatens resource-constrained agricultural production systems worldwide. Genomics-assisted breeding (GAB) approaches have greatly contributed to enhancing crop breeding efficiency and delivering better varieties. Fast-growing capacity and affordability of DNA sequencing has motivated large-scale germplasm sequencing projects, thus opening exciting avenues for mining haplotypes for breeding applications. This review article highlights ways to mine haplotypes and apply them for complex trait dissection and in GAB approaches including haplotype-GWAS, haplotype-based breeding, haplotype-assisted genomic selection. Improvement strategies that efficiently deploy superior haplotypes to hasten breeding progress will be key to safeguarding global food security.

## Abstract (web summary)

In this Review, Bhat et al. highlight ways to mine crop haplotypes and apply them for dissecting complex traits and genomics-assisted breeding (GAB) approaches. This Review presents new avenues to discover superior haplotypes and assemble them in targeted manner in crop breeding for faster delivery of high-yielding cultivars with better adaptation to future climates.

## Introduction

Crop plants are subjected to a variety of biotic and abiotic stresses that impair normal crop growth and cause substantial losses in crop yields worldwide^1,2^. Amid these stresses, developing climate smart and nutritious crop varieties that remain vital to securing food security of the incessantly growing human population, presents a daunting challenge to the agricultural scientists worldwide. Although conventional breeding has made great success in the development of high-yielding crop varieties^3^, it is important to accelerate the pace of crop improvement programmes especially for the complex traits such as yield under stress conditions. In this regard, the genomics-assisted breeding (GAB) by implementing genomics tools in breeding was proposed by Varshney et al.^4^. This approach has delivered several high-yielding, stress-tolerant and better nutrition varieties^5,6^. For instance, the low-throughput sequence-based markers, such as simple sequence repeats (SSRs), were extensively used in the molecular breeding programmes; however, these marker systems have limitations such as low density across the genome, low coverage, expensiveness. Application of these second-generation DNA marker systems resulted in poor resolution of gene mapping and relatively low efficiency of plant selections and breeding^7,8^. Fortunately, recent advances in the next generation sequencing (NGS) and the genotyping platforms have considerably alleviated this bottleneck in crop breeding. These NGS-based platforms have provided remarkable marker-density and coverage at reduced cost^9^, and are now commercially available for both model and non-model crop species^10,11^. These high-throughput platforms make hundreds of millions of DNA polymorphisms accessible for use in genetic and genomics research^12,13^; and their application in crop breeding has considerably increased the gene mapping resolution and prediction accuracy in genomic selection (GS)^14,15^. Majority of the economically important crop traits, such as yield, quality and stress tolerance, are of complex quantitative nature, which are influenced by several small effect QTL/genes and manifest substantial genotype x environment (G x E) interactions^16^. Although efforts to understand the complex genetic makeup of these agriculturally relevant traits have been successful in the identification of major-effect genomic regions, conventional experimental populations faced the problem of limited genetic diversity, low resolution and limited recombination events^17,18^. Hence, the genome-wide association study (GWAS) has emerged as a powerful tool for dissecting complex quantitative traits in crop plants with enhanced resolution and allelic richness^19,20^. Furthermore, due to the availability of cost-effective and high-density genotyping platforms, it has been possible now to screen larger breeding populations for estimating and using the breeding value in crop improvement programmes by using GS, another breeding approach^21^.

In recent years, the NGS-based genotyping methods such as genotyping-by-sequencing, restriction site-associated DNA sequencing, whole-genome resequencing as well as fixed SNP arrays have greatly facilitated genotyping of large germplasm collections for GWAS and GS analyses^8,22^. However, the major limitations for the use of SNPs in these analyses include their biallelic nature, the presence of rare alleles, and abundant levels of linkage drag^16,23^. Therefore, the candidate genomic loci identified by GWAS often do not represent the causative locus; but correspond to the loci that are in linkage drag with a gene or a regulatory element, eventually affecting the trait of interest^24,25^. In this regard, an effective approach to overcome the limitations of SNPs and increase the resolution of candidate genomic regions is to consider haplotypes for genome-wide analyses^26^. Haplotype is a specific combination of jointly inherited nucleotides or DNA markers from polymorphic sites in the same chromosomal segment^27,28^.

In the present review, we discusses the potential and need of haplotypes in the crop breeding for the development of improved varieties. We have also compared the efficiency of haplotype- and individual SNP-based markers in the GWAS and GS analyses. Besides, the challenges associated with the use of haplotypes in crop breeding at the commercial level are also addressed. We conclude by highlighting the scope of haplotypes in the future crop breeding programs.

### Crop improvement: conventional breeding to genomics-assisted breeding

Development of improved crop varieties for food, feed and industrial purposes can be accomplished mainly by plant breeding^29^. The science of plant breeding has evolved from conventional to present day GAB^6,30^. In the last century, tremendous efforts have been made by plant breeders across the globe to develop improved varieties in different crop species by using the conventional breeding approaches^31–45^. It is estimated that the undernourished proportion of the human population has been reduced from 40% in the 1960s to <11% now, which is principally attributable to the improved high-yielding and stress-tolerant crop varieties produced mainly through conventional breeding^44^. The conventional plant breeding for crop yield enhancement progressed consistently over time. The high-yielding varieties/hybrids were mostly responsible for this increase in both area and productivity, and the large-scale adoption of these varieties/hybrids provides strong evidence for contributions by plant breeding innovations over the last century.

In recent years, the plant breeding community has recognized the need of introducing genetic variability in breeding programs to enhance the genetic base of elite gene pool, enhancing precision and efficiency in selection and reducing the breeding cycle^4,6,46^. In this context, the GAB approach proposed by Varshney et al.^4^ outlined the use of genomics tools and technologies to identify markers, candidate genes associated with target traits and integration of genomics approaches in breeding. Several GAB approaches including marker-assisted backcrossing (MABC), marker-assisted selection (MAS), marker-assisted recurrent selection (MARS) and advanced backcross QTL (AB-QTL) were suggested for crop improvement. In recent years, GS approach has also been added to GAB portfolio^6,21^. For MAS, the first step is the identification of molecular markers that are strongly associated with genomic regions/quantitative trait loci (QTLs) regulating the traits of interest. Eventually, these QTLs, either individually or in multiple numbers, can be pyramided into elite breeding material through MABC. Some success stories of MABC include the introgression of a *‘QTL-hotspot’* into elite chickpea varieties for improved yield under drought conditions^47,48^, improving the yield and stress tolerance of mega rice variety IR64 (Developed by IRRI, IR 64 was released in Phillipines in 1987. The rice variety registered a widespread acceptance owing to its multiple beneficial traits including better cooking quality, earliness, disease resistance and high yield)^49,50^, transferring QTLs (*qDTY2.2* and *qDTY4.1*) into IR64 for reproductive stage drought tolerance^51,52^, and the improvement of different yield and stress-related traits in several major crop species^6,53–55^. Despite the aforementioned utilities of MABC, it is efficient only for the major-effect QTLs, while most of the genetic variations for yield, quality and stress tolerance traits in crop plants are governed by a large number of minor QTLs. Alternatively, the frequency of many beneficial alleles can be increased in a given population through the MARS scheme. Unlike MABC, the MARS has been applied for improving a breeding population with respect to QTLs exerting smaller effects on the phenotype. MARS has been successful in improving drought tolerance in multiple crop species viz., maize, soybean, sunflower, wheat, sorghum, and rice^56–60^. To capture minor effect QTLs scattered throughout the genome, the plant breeding community has recently started to use GS approach. GS estimates the genetic worth of an individual based on the large set of marker information distributed across the whole genome, rather than a few markers as in the case of MAS^21^. In this approach, a prediction model based on the genotypic and phenotypic data of training population (TP) is developed and then genomic estimated breeding values (GEBVs) for the individuals of breeding population (BP) are computed from their genome-wide marker profiles^61^. The GEBVs allow one to predict individuals that will perform better and are suitable either as a parent for the next breeding cycle or can directly enter into the variety release pipeline^21^. Unlike MAS, GS does not necessarily require a prior knowledge of significant marker-trait associations^62^. However, inclusion of the significant set of markers, such as resulting from GWAS, into GS models has been found to improve prediction accuracies^63^. GS has started gaining profound interest in plant breeding, with the recent studies establishing its superiority over other selection methods^64–70^. With the availability of a range of cost-effective genotyping platforms and advances in the development of prediction models, GS is expected to be a routine breeding approach, like MABC/MAS in crop improvement programmes.

### Features of haplotypes

#### Defining haplotypes: harnessing the wealth of whole-genome sequencing data

**Fig. 1:**
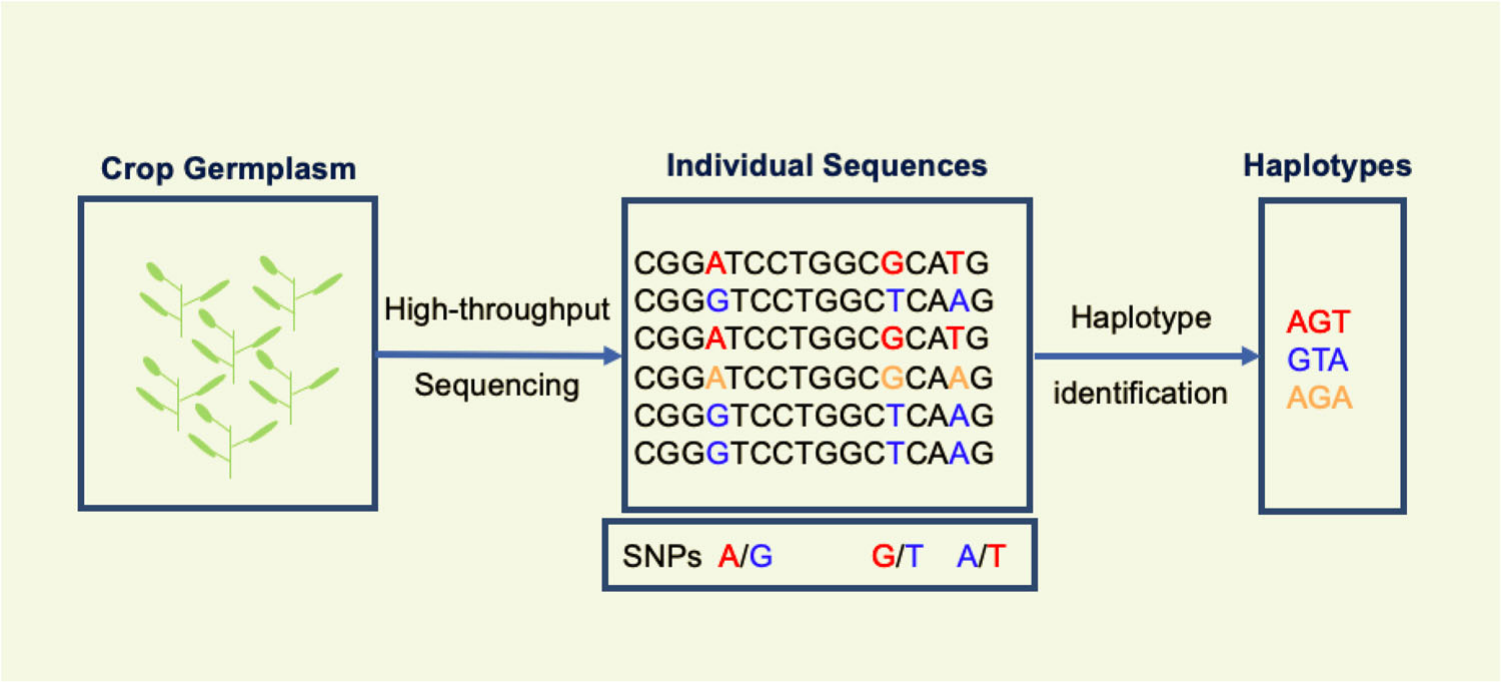
Formation and development of haplotypes from haploid sequences. Resequencing of the crop germplasm is done to identify the polymorphic SNPs to be subsequently used in the development of haplotypes.

Haplotype is a combination of alleles for different polymorphisms (such as SNPs, insertions/deletions and other markers or variants) present on the same chromosome, which are inherited together with minimum chance of contemporary recombination^71,72^. Any individual has two haplotypes for a given stretch of chromosomal DNA; while at the population level, many haplotypes can be found for the same stretch^73^. In other words, a haplotype is defined as a set of nearby genomic structural variations, such as polymorphic SNPs, with a strong linkage disequilibrium (LD) between them^74^. As shown in Fig. 1, two or more polymorphic SNPs of the haploid sequences inherited together as a unit constitute a haplotype^71^. The haplotypes are defined/assigned in three principal ways: (a) by using the haplotype diversity in a given chromosomal segment, (b) by using the pairwise LD between the jointly inherited markers that show lack of evidence for historical recombination, it is measured by *r*^*2*^ (measure of LD)^75,76^ and (c) by grouping of SNPs through sliding-windows of fixed or variable length^77^. Evidence suggests that the LD-based approaches are more efficient for defining the haplotypes in the genomic/chromosome regions^26,74^. This is because (a) historical recombination identification is the direct focus in a particular population through the haplotype detection, (b) visualization of the LD coefficients is very easy, (c) for diploid data with unknown haplotype phase, it is applicable. The LD in the given population is determined by many factors such as mode of pollination, population size and structure, mutation rate, genetic drift, recombination frequency, and the type of selection on a given chromosomal fragment^78^.

During the evolution of the important crop species such as rice, maize, wheat, sorghum, cassava and rapeseed, the selection of genes/alleles regulating desirable phenotype for the trait of interest is the major factor responsible for the formation of signatures of selection^26^. The signatures of selection (also known as conserved haplotype blocks and selective sweeps) possess multiple genes, which are regulated together by many regulatory genes. The correlation among different traits as reflected from the selection signatures is either due to the true linkage among the genes or resulting from the pleiotropic effect of the same genes^34,79^. Therefore, the crop breeders should preferably target these genomic regions to elucidate their effect on the traits of interest. Besides, the integration of genomics to identify the recombinants produced by crossing of contrasting parents will greatly assist in resolving the complexity of quantitative traits. This will enhance the efficiency to improve the specific traits in modern varieties for their better adaptation to extreme environments^80^.

Due to the availability of sequencing data from large number of individuals for a given crop species it has been easier to define the haplotype. By using the whole genome sequencing data, Bevan et al.^81^ defined the concept of the haplotype assembly. Together with the phenotyping data of germplasm/breeding lines, it is possible to assess and validate phenotypic effects of the ‘component’ haplotypes. Based on this premise, and by using large-scale whole-genome resequencing datasets in combination with *haplo-pheno* analysis, Abbai et al.^82^ identified useful haplotypes for future breeding in rice and Sinha et al.^46^ followed the similar approach in pigeonpea. High-density SNP data generated from multiple genotypes via NGS-based or array-based approaches have been used for the development of haplotypes in many plant species. These haplotypes have also been used for various applications in research and breeding in different crop species (see details in Tables 1, 2).

**Table 1:** The use of haplotype markers in genome-wide association mapping (GWAS) analyses in different crop species.

Crop Species	Trait	Population size	Haplotype markers	Haplotype-trait associations	PVE (%)	Reference
Soybean	100-seed weight; plant height; seed yield	169	941	87	9.14-15.83	^134^
Soybean	Agronomic and yield-related traits	296	–	10	>10.0	^153^
Wheat	Heading date; plant height; 1000-grain weight; grain number per spike; fruiting efficiency at harvest	102	4516	97	–	^121^
Wheat	Grain yield; days to heading; plant height	6461	519	36	2.2-5.6	^154^
Barley	Deoxynivalenol content in kernels; heading time; days to maturity; grain yield; plant height; specific weight; 1000-kernel weight	277	14,400	–	2.0-14.0	^135^
Barley	Yield and quality-related traits	106	2770	23	>10.0	^131^
Rice	Grain shape	372	–	30	–	^155^
Rice	Agronomic traits	414	15,275	–	–	^109^
Maize	Agronomic and reproductive traits	322	53,403	44	5.6-17.0	^156^
Maize	Total plant height; ear height; ear height/plant height	183	7,831	40	7.0-22.0	^74^
Maize	Agronomic and reproductive traits	>1000	154,104	–	>10.0%	^157^
Oat	Heading date	4657	164741	184	–	^158^
Rapeseed	Days to flowering; seed glucosinolate content	950	–	15	–	^152^

### Third-generation sequencing: alleviating the bottlenecks in haplotype identification

The long-term goal of genetics is to elucidate the effect of DNA sequence variations on the plant traits, and how these variations have led to the evolution of different populations and species^83,84^. In genetics, linkage is a core concept on which molecular mapping of genetic determinants relies. For example, in the linkage or association mapping, the individual genetic markers/variants are used to determine their association(s) with the trait(s) of interest, instead of pinpointing the causal mutation^3^. The trait-associated DNA markers are then used as surrogates for the selection of the desirable phenotypes^5^. As we mentioned in the previous section, fast-tracking the process of targeted trait improvement will require a paradigm shift from individual SNP markers to haplotypes. The information on haplotypes regulating the important phenotypes is currently limited in the genetic studies^85^, which prevents the accurate determination of ancestry reconstruction, rearrangements of chromosomes, allele-specific expression, and detection of selective sweeps^86,87^.

However, the availability of the high-throughput sequencing platforms has made a tremendous impact on the identification of haplotypes and their application in the genetic studies. Although, the second-generation sequencing techniques produce short reads of 150 bp, these small reads normally do not possess more than a single variant^88^. Hence, the haplotypes are constructed indirectly from this data and this needs specific statistical inferences from population genotyping data, which in turn increases the time and cost for the haplotype construction^88,89^. In contrast, third-generation sequencing (TGS), such as the Pacific Biosciences (PacBio) and Oxford Nanopore Technologies (ONT), produce long reads from which the haplotypes can be directly constructed^88^. In comparison to the second-generation sequencing methods, analysis of DNA molecules can be performed directly via long-read sequencing platforms^90^. However, the ‘phasing’ is used for some adjustment of the long-read sequencing data to increase the efficiency for haplotype identification. Construction of the haplotypes from the sequence data through haplotype estimation is known as phasing; which is very important to elucidate the sequence-specific variations such as the effect of methylation, specific expression of alleles and compound heterozygosity^91^. Fixing of higher error rate (~10%) in the long-read sequencing technologies compared to short-read sequencing methods (NGS methods) needs specific bioinformatics-mediated adjustments^92^. In this regard, many different phasing methods enabling haplotype construction/reconstruction from long-read sequencing data have been recently developed, such as reference-based phasing (molecular haplotyping, single-cell phasing, and polyploid phasing), *de novo* genome assembly (such as diploid and polyploid assembly) and strain-resolved metagenome assembly (de novo re-assembly, single nucleotide variant-based assembly, read and contig binning)^72^. Combination of these haplotype analysis methods with various computational tools such as WhatsHap, HapCut2, HapTree, WhatsHap- polyphase, Falcon phase, Hifiasm, SDip, POLYTE, DESMAN, MetaMaps, and ProxiMeta, has greatly enhanced the efficiency and precision in the identification of do novo and rare variants from the long-read sequencing data^72^. Therefore, integrating the various phasing and bioinformatics tools with the long-read sequencing technologies has allowed us to fully exploit the potential of these sequencing approaches in haplotype construction^91^. For example, Ammar et al.^73^ showed that MinION nanopore sequencer efficiently resolved the variants/haplotypes of *HLA-A*, *HLA-B* and *CYP2D6* genes by producing the long reads without even using the statistical phasing. Similarly, Zhang et al.^93^ also demonstrated the higher accuracy of Nanopore sequencing in the identification of haplotypes across the genomes. Besides, recent advances in the PacBio’s HiFi technology have allowed to produce long reads in the range of 15-20 Kb, with an error rate comparable to the second-generation sequencing i.e., more than 99% accuracy was achieved^94^. These advancements have allowed reconstruction of the previously impossible near-complete human haplotypes that include microsatellites, repetitive elements, and other complex structural variations^95^. Moreover, Sun et al.^96^ used the PacBio HiFi reads (30x per haplotype) and hifiasm to produce the assembly of the autotetraploid genome of potato. This was the first study demonstrating the haplotype-resolved assembly of potato crop. Through single-cell genotyping and high-quality long-read sequencing of the tetraploid plants, the authors successfully reconstructed all four haplotypes showing considerably higher diversity among themselves. This haplotype diversity is significantly higher than the diversity commonly found within a given species. This evidenced that successful haplotype reconstruction in the polyploid species has a huge impact on breeding these crops in the future^96^. Recent research demonstrates the enormous potential of the TGS in resolving the accuracy issues in the haplotype identification, thereby increasing the scope of haplotypes for genetic studies in both animals and plants^72^. Hence, the TGS platforms offer promising alternative to obtain haplotype-related information from the genomes, and future affordability of these sequencing platforms will have a profound impact on plant research and breeding.

#### Haplotagging: A novel sequencing strategy for rapid discovery of haplotypes

Recently, a simple, rapid and promising technique for linked-read (LR) sequencing (called ‘haplotagging’) has emerged^97,98^. In this technique, molecular barcoding of long DNA molecules is carried out prior to sequencing, which in turn retains the long-range information by preserving the linked variants^85^. The shared barcode is then used to link the individual short reads for constructing the original haplotype^98^. However, currently the commercial utilization of haplotagging in the genetic studies is prevented by certain factors, which include the requirement of custom sequencing primers, and cost-ineffectiveness, and poor scalability of the current techniques^98^. Nevertheless, if managing these factors, especially the lower cost and more scalability, becomes possible in near future, the haplotagging will be greatly used in the genetic studies. For instance, it will enable the haplotyping of the larger plant and animal populations, and allow the sequencing and systematic discovery of haplotypes in tens of thousands of samples, that too in both model and non-model plant species. It has been documented that both standard Illumina sequencing and haplotagging maintain full compatibility, and there is no extra cost in the haplotagging^98,99^. The utility of haplotagging technique, for the identification of the haplotypes in the genome, has not yet been demonstrated in the plants, but recently, the haplotagging has been demonstrated in the two butterfly species^85^. For example, Meier et al.^85^ applied haplotagging approach to generate the haplotypes of megabase-size for the case of around six hundred butterflies’ individuals belonging to the two species viz., *Heliconius erato* and *H. melpomene*, and these two species were identified to form hybrid zones that are overlapping across an elevational gradient in Ecuador. Besides, Meier et al.^85^ also showed that haplotagging was able to detect the genetic loci regulating the distinct wing color patterns, namely, high- and low-land. In both the species the different haplotype alleles were detected at the same major loci; however, the chromosome rearrangements show no parallelism. To this end, this study demonstrated that technique of the “haplotagging” was successful to identify the distinct haplotype allele classes regulating the different phenotypes of the wing color patterns. Hence, these results suggested the enhanced power of the efficient haplotyping methods when combined with large-scale sequencing data from natural populations^85^.

The above findings suggest the potential role of haplotagging in the identification of haplotype alleles regulating different phenotypes for a particular trait of interest. Hence, the haplotagging technique might be a promising strategy to identify the superior haplotype alleles in the diverse plant populations/germplasm for their ultimate use in the breeding for the development of improved crop varieties. This technique will be crucial to harness the true potential of the haplotype-based breeding for crop improvement.

#### Haplotype vs. individual markers: Comparative efficiency for crop breeding

Variations in the complex phenotypes are associated with the presence of SNPs, insertion–deletions and copy number variations in certain genomic loci^100–102^. Currently, most of the plant breeders are using SNP markers to tag novel genetic variations underlying different phenotypes, and introgress these variations into the elite crop cultivars. However, the superiority of haplotype markers compared to individual SNP markers in addressing complex traits has been demonstrated through efficient gene identification and GS^26^. For example, the use of haplotypes has been reported to considerably increase the prediction accuracy of the low-heritable quantitative traits as compared to the individual SNP markers^103–107^. Besides, the use of haplotypes in gene mapping analyses has emerged as a more efficient approach for the identification of genomic loci and candidate genes regulating traits of interest^72,108,109^. The latest evidence suggests that the haplotype-based approach can improve not only the predictive abilities of GS models but also the precision with which genomic loci are detected in GWAS^109–111^.

The higher efficiency of the haplotypes over individual SNP is due to some important reasons. For example, SNPs tiled on arrays are usually chosen for their moderate to high minor allele frequency (MAF). Therefore, most of the SNPs in the commercial chips are expected to be the old mutations, given that all new mutations remain at a low frequency in the beginning and a large part of them may disappear before reaching considerable frequency^112^. Since the single-nucleotide-based genomic relationship matrix (G_SNP_) is based on SNPs with relatively high MAF, this may imply that G_SNP_ traces old relationships from distant relatives and, therefore, may trace less accurately the changes due to recent selection as compared to the multi-locus haplotype-based relationship matrix, G_HAP_^112^. Meuwissen et al.^112^ suggested that building the relationship matrix using haplotypes instead of single SNPs may improve the accuracy of genomic predictions. Another potential limitation of G_SNP_ is that the SNPs are biallelic and, therefore, their polymorphism information content (PIC) value is not high. This restricts the ability to effectively capture LD between SNPs and multi-allelic QTLs. On the other hand, haplotype blocks are generally “multi-allelic” and may therefore better capture LD with multi-allelic QTLs compared to individual SNPs^112^. It is also worth noting that longer haplotype blocks provide more information about possible recent mutations and close relationships than the shorter ones^113,114^. Furthermore, haplotype effects could also factor in local epistatic effects among QTLs located within the haplotype blocks^113^. In addition, G_HAP_ can differentiate between identical by descent (IBD) and identical by state (IBS), while G_SNP_ cannot. This is because long shared haplotype blocks are likely to come from common ancestors. Therefore, long haplotype blocks can better capture information on IBD regions than individual SNPs in GS experiments^115^.

### Applications of haplotypes in genetic analysis and breeding

#### Gene mapping

Recent studies elucidate the great potential of GWAS for the genetic dissection of important traits in major crop species. Researchers have mostly used SNP markers for the GWAS analysis^116^, because of the ability of the NGS-based genotyping systems to provide genome-wide marker data in cost- and time-efficient manner^11^. As mentioned earlier, SNP markers are biallelic in nature having low informativeness and mutational rate^117^. Besides, the SNP arrays possess the inherent ascertainment biases, and thus in the GWAS analyses, the significant SNPs often do not represent the causal molecular variants^5,8^. It can be explained by the fact that rare alleles often determine the extreme phenotypes^23^. The existence of LD between true molecular variant and the non-causative markers causes stronger marker-trait linkage than that of causal variant itself^25,118^.

**Fig. 2:**
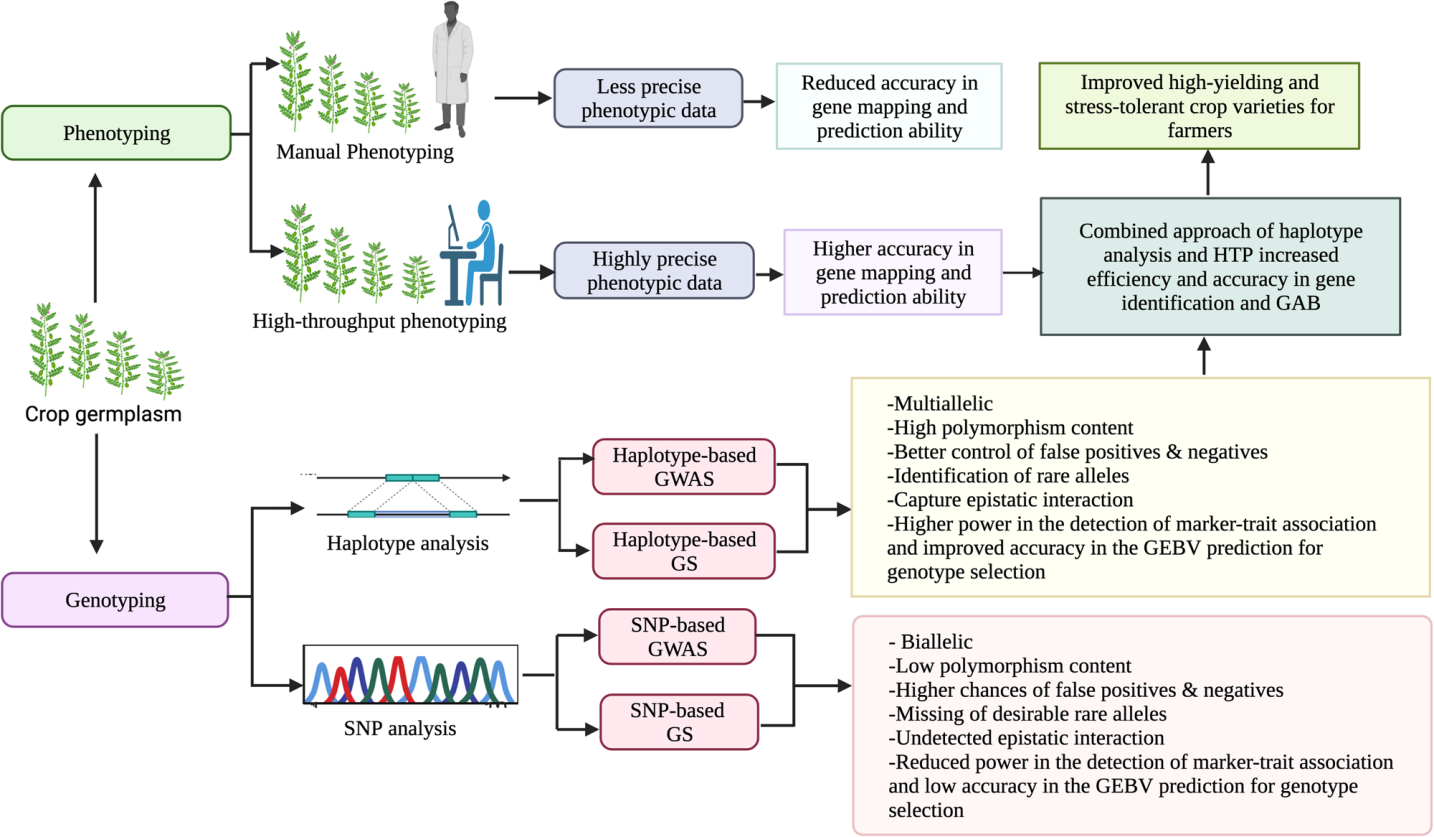
Mining of SNPs and construction of haplotypes for detecting marker-trait associations (GWAS) and computing genomic estimated breeding values (GS). This diagram describes the comparative potential of the Haplotype-Based GWAS/Haplotype-Based GS in relation to SNP-Based GWAS/SNP-Based GS for the development of improved crop cultivars via genomics-assisted breeding (GAB). It showed that Haplotype-Based GWAS/Haplotype-Based GS in combination with the high-throughput phenotyping (HTP) has great potential to enhance the precision and accuracy in the gene identification and GAB. The image was created using BioRender (https://biorender.com/).

Several researchers advocate for using haplotypes for conducting GWAS (Fig. 2). Recent GWA studies based on empirical and simulation data have revealed higher mapping accuracy and power of haplotype blocks over individual SNPs for the detection of QTLs/genes^76,119–122^. A variety of reasons explain this superiority of haplotypes (Fig. 2). For example, Stephens et al.^27^ demonstrated that the multi-allelic nature of haplotype blocks makes them more informative compared to SNP markers (biallelic in nature). The authors reported higher abundance of haplotype variants than SNPs, indicating recombination and recurrent mutation events within and among the genes in the haplotype. Moreover, the haplotype-based analysis is expected to control false positives and reveal the complex mechanism of causal haplotypes in a better way as compared to individual SNPs. For example, the repulsion states between two causal QTLs located close to each other^26^. In particular, haplotype-based analysis can capture epistatic interactions between SNPs at a locus^123,124^, provide more information to estimate whether two alleles are IBD^125^, assess the biological role played by neighboring amino-acids on a protein structure^123^, reduce the number of tests and hence the type I error rate^126^, capture information from evolutionary history^127^, and can provide more power than single marker system to analyze an allelic series existing at a particular locus^128–131^. To this end, Hamblin and Jannink^129^ reported that as compared to individual-based SNP markers, the haplotype approach increased the allelic effect and phenotypic variation explained (PVE) by 34% and 50%, respectively. N’Diaye et al.^120^ observed that by combining multiple SNPs into haplotype blocks, the average PIC increased from 0.27 per SNP to 0.50 per haplotype in wheat. Over the last few years, haplotype-based GWAS analyses have identified important QTLs and candidate genes for various crop traits (Table 1). Greater power of haplotype-based mapping compared to SNP-based GWAS in the detection of genetic loci associated with the plant height and biomass was evident in maize^119^. It is interesting to note that in comparison to single SNP-based mapping the haplotype-based mapping detected fewer significant associations and candidate genes for drought tolerance in maize; however, with higher PVE values^132^. Recently, applications of haplotype-based GWAS for various traits including yield, quality and stress tolerance in different plant species such as Arabidopsis^133^, soybean^134^, wheat^121^, barley^131,135^, rice^136^ and maize^137^ have shown great promise for trait discovery and crop improvement.

However, the presence of non-informative SNPs in a given haplotype block (either small or long block) masks the effect of adjacent informative SNPs, which in turn leads to spurious associations, decreasing the effectiveness of the GWAS analysis^138^. Hence, the haplotype-based GWAS and GS analyses uses the approaches such as sliding windows of fixed/variable length, haplotypes diversity among samples, LD between adjacent SNPs, and SNP number within haplotype to construct the haplotype blocks^139^. All these approaches have one thing in common i.e., they all use the consecutive SNPs that possess high LD for the development of haplotypes. Therefore, under many circumstances, the haplotypes generated via these approaches’ have been observed to show no difference in the information provided by the haplotype and single SNP, because the SNPs in high LD provide redundant information^140^. To this end, recently a new haplotype-based GWAS approach called FH-GWAS has been introduced^76^. This approach uses a different method to generate haplotypes i.e., only those SNPs are combined into functional haplotypes that possess true contribution to the haplotype effects via additive and/or epistatic effects. Thus, FH-GWAS is able to overcome the constraints of combining redundant SNPs (in high LD) into haplotypes and avoids the highly time-consuming process of selecting optimal combinations of SNPs. It is therefore expected to be more powerful than SNP-based and other haplotype-based GWAS approaches.

#### FH-GWAS analysis: an efficient substitute for discovering superior haplotype alleles

Notwithstanding the superiority of GWAS based on haplotypes over SNPs, the use of haplotypes in the GWAS faces some challenges^141^. For instance, the contrasting effects of different haplotype allele classes will be diluted if the irrelevant markers are added to a possible causal genetic variant^123^. Theoretically, in the case of a haplotype with *m* SNPs, the total number of different haplotype alleles will be equal to 2 ^*m*^. This will increase the degree of freedom (this holds good for the estimation of population structure but not for GWAS, especially in the estimation of means and variance if the haplotypes are identified only once or twice), and that in turn will diminish the power of association analysis^131^. However, the 2^*m*^ formula for determining the number of haplotype alleles do not always work in practice because haplotype diversity is affected by a variety of factors including genetic structure and size of the population, mutation, recombination, marker ascertainment and demography^142^. For example, Scott et al.^143^ by analyzing a panel of 16 wheat genotypes, representing the founders of MAGIC population, established that by using the SNPs of the promoter and genic regions, at most of the genes no greater than three haplotypes are identified, and most of the genes were biallelic. Besides, the most critical factor affecting the haplotype-based GWAS analysis is the method(s) used for the construction of haplotypes, as discussed in the previous section. Only the consecutive SNPs in high LD are grouped into the haplotypes in all these methods. Sometimes the redundant information is provided by the SNPs that are in high LD, and as a result the use of these haplotypes does not provide more information than the individual SNPs^140^. This explains the contradictions reported in recent studies regarding the efficiency of haplotype- and SNP-based GWAS approaches^76^. As discussed above, the alternative approaches have been proposed for the identification of the haplotypes with non-consecutive SNPs that provide more information than the haplotypes with consecutive SNPs^74,140,144^. Also, high computational burden associated with these approaches, further limits their use in the association studies^74^.

**Fig. 3:**
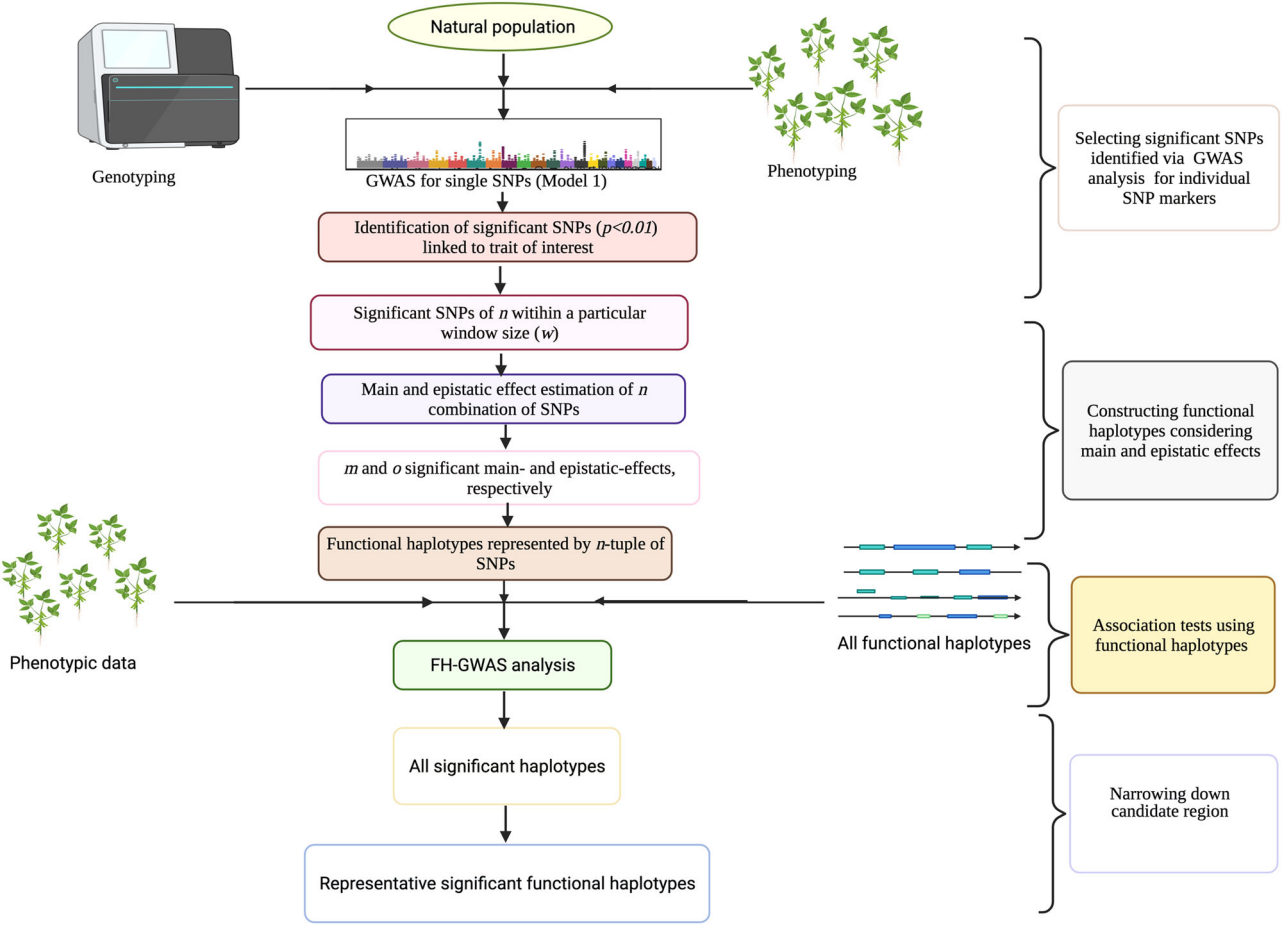
Functional haplotype-GWAS (FH-GWAS) analysis for identification of the superior haplotypes for traits of interest. FH-GWAS approach first involves the individual SNP-marker based GWAS analysis (Model 1), that allows the identification of the candidate SNPs (SNP-trait association). This step is followed by the identification of the closely linked adjacent SNPs within a specific range in a chromosome region; and the SNPs within this specific region possessing additive and/or epistatic effects as well as have true contribution to the haplotype effects are combined into the functional haplotype. Lastly, the GWAS analysis was again performed by combining the functional haplotypes and phenotypic data, that ultimately leads to the identification of significant haplotypes associated with the trait of interest. The image was created using BioRender (https://biorender.com/).

To alleviate the limitations of the haplotype-based GWAS, an alternative efficient approach based on functional haplotype-based-GWAS (FH-GWAS) has been introduced to identify the superior haplotype alleles for the trait of interest^76^ (Fig. 3). Given the significant role that the epistasis plays in the regulation of complex trait variations, FH-GWAS takes the associated epistatic effects of SNPs into consideration for trait discovery^24,145,146^. Hence, in FH-GWAS, the SNPs possessing mild threshold for the main effects are first selected, followed by the identification of consecutive and/or non-consecutive combinations of SNPs (having significant epistatic effects) in a chromosomal region of defined size (Fig. 3). This approach combines only those SNPs into a functional haplotype that really contribute to the haplotype effects via additive and/or epistatic effects, thus preventing the redundant SNPs (with high LD) from combining into a haplotype. Besides, it prevents the laborious and time-consuming search for the detection of the optimal combinations of SNPs. In this regard, FH-GWAS is more powerful and efficient compared to haplotype-based and SNP-based approaches.

FH-GWAS outperformed SNP-based approach in a simulation study unless the SNPs of the haplotypes possess low MAF and the LD of haplotype SNPs is high^76^. Analysis of flowering-time trait in a large population of *Arabidopsis thaliana* using FH-GWAS has revealed its great potential and efficiency in the association studies^76^. Importantly, FH-GWAS detected all the genomic/candidate regions that were also identified via the SNP-based and haplotype-based GWAS approaches; however, it was only the FH-GWAS that could find a novel genomic region for flowering time on chromosome 4 of *A. thaliana*^76^. In view of the evidences available from both simulation and empirical studies, FH-GWAS arguably holds a great promise for trait mapping in crop breeding. Further, this approach can be used for any crop species, particularly the homozygous ones, where sufficient coverage and suitable size of SNPs are available^76^. However, if the FH-GWAS is to be used for the improvement of multiple traits, the construction of functional haplotypes for each individual trait must be done separately, as the tests of main and epistatic effects of markers are trait-dependent. Although FH-GWAS can improve the efficiency of the gene-trait association studies, this approach is computationally demanding in comparison to the other haplotype-based approaches^76^.

#### Haplotype-based breeding (HBB)

The development of stress-tolerant crop varieties with improved yield potential is one of the major challenges for breeders, especially in the face of global climate change^3,124^. As discussed earlier, GS has emerged as an efficient approach for addressing complex polygenic traits, population improvement and developing improved varieties. The germplasm pool of the most crop species possesses complex genome structure; hence, the use of haplotypes in GS has been proposed as a powerful approach to improve the accuracy and efficiency in the prediction ability^26^. This is because the comprehensive haplotype maps allow the identification and utilization of genomic regions linked to a particular trait at higher accuracy in populations with pronounced LD structures^4^.

Implementation of haplotypes in crop improvement is accomplished through two approaches, viz., retrospective and prospective^81^. During the long-term selection process, the plant breeders have selected the favorable haplotypes that lead to desirable phenotype(s) for the trait(s) of interest. Hence, by using the genome resequencing approach to sequence an elite gene pool, these favorable haplotypes can be identified in the elite crop germplasm^26^. Furthermore, the molecular markers that define these favorable haplotypes can be developed and then all these haplotype-defining markers can be used to select the most desirable combination of haplotypes governing the specific phenotype. Besides, these haplotype-related markers can be used to separate favorable and unfavorable genetic variation by identifying lines with novel recombination in chromosomal blocks of interest. On the other hand, the haplotypes can also be used in the prospective manner, in which the large collection of ancestral and wild germplasm of particular crop species (not only the elite breeding pools) can be re-sequenced to identify haplotypes with a broader range of genetic variation^81^. In this approach, the genome-wide haplotypes are used to identify the novel haplotypes present in the wide range of natural germplasm. Hence, the main objective of this approach is to identify the new, desirable and superior haplotypes. In summary, based on information/utility of various haplotypes, it is possible for assembling desirable haplotype combinations to develop optimal parents in breeding programmes. Deployment of haplotypes in breeding as mentioned above has been referred as haplotype-based breeding (HBB)^6,20^.

#### Haplotype-assisted genomic selection

The prediction accuracies of GS models for yield and stress-related traits have outperformed the classical selection models, implying that GS is particularly suitable for the improvement of high-yielding and stress-tolerant crop cultivars^3,147^. For example, Zhang et al.^148^ demonstrated higher prediction accuracy of GS (0.75–0.87) as compared to MAS (0.62–0.75) for important agronomic traits in soybean. Similarly, GS was found superior to phenotypic selection for improving multiple agronomic traits related to yield and stress tolerance in different crop species^147^. Besides, GS can reduce the time required to complete a selection cycle in crop plants, which can lead to increased production of the commercially important crops^7,149^. Because of their high PIC value, fitting haplotypes with statistically significant associations to phenotypes as fixed effects in GS models could further improve prediction accuracies^150,151^. The haplotype-assisted GS depicts the complex relationships between genotypic information and phenotypes more accurately than individual SNPs. Hence, this approach could ultimately help further increasing selection gain per unit of time. The use of haplotypes may improve the accuracy of genomic prediction because haplotypes can better capture LD and genomic similarity in different lines and may capture local high-order allelic interactions^109^. Additionally, prediction accuracy could be improved by portraying population structure in the calibration set. A recent GS study that compared the prediction ability computed from haplotypes and SNPs in a set of 383 advanced lines and cultivars of wheat established the superiority of haplotype-based predictions over SNP-based predictions for all studied traits i.e., yield, test weight and protein content^152^. As compared to the individual SNPs, the combined use of haplotypes of 15 adjacent markers and training population optimization significantly improved the predictive ability for yield and protein content by 14.3% (four percentage points) and 16.8% (seven percentage points), respectively. Similar results were reported by other researchers in different crops such as maize^151^, *Brassica napus*^152^, and sorghum^80^. Recent examples on the use of haplotype markers for genomic selection/prediction analysis in different crop species are presented in Table 2. Taken together, these studies underscore better performance of haplotypes in comparison to individual markers in improving prediction accuracies of GS for complex traits. Hence, the use of haplotypes in GS will definitely increase the prediction ability and greatly assist in harnessing the true potential of GAB in crop improvement.

**Table 2:** The use of haplotype markers in genomic selection in different crop species.

Crop Species	Trait	Training Population size	Haplotype markers	GS prediction accuracy	Reference
Bluegum	Traits related to wood quality and tree growth	646	~3000	0.58	^105^
Soybean	Plant height & grain yield per plant	235	357	>0.80 & >0.45	^159^
Sorghum	Agronomic and yield-related traits	207	1,974	0.57-0.73	^160^
Wheat	Yield, test weight, and protein content	383	1400	>0.40	^151^
Wheat	Grain yield and related traits	4,302	1162	0.39-0.48	^154^
Oat	Heading date	635	13954	0.42-0.67	^158^

## Conclusion

GAB approaches aim to accelerate the pace of genetic gain and contribute to the global food and nutrition security. Several GAB approaches such as MABC, MARS and more recently GS have been successfully utilized for developing superior varieties. However, in the context of large-scale genome resequencing projects of germplasm accessions and breeding lines, it is possible to define new haplotypes. The availability of long-read sequencing technologies is also accelerating the discovery of haplotypes that are helpful to improve genome assembly. From applications perspective, these haplotypes can be used for a variety of purposes. Instead of using SNPs, haplotype-based GWAS analysis identifies causal polymorphism in a precise manner. Similarly, evidence demonstrating higher genomic prediction efficiency, based on haplotypes as compared to SNPs, encourages researchers to increasingly embrace haplotypes-assisted genomic prediction in crop improvement programmes. Furthermore, advances in high-throughput phenotyping would enhance discovery and subsequent applications of superior haplotypes in crop breeding. We believe that haplotype-based research and their applications will be routine to develop improved cultivars for future food security.

### Reporting summary

Further information on research design is available in the Nature Research Reporting Summary linked to this article.

## Supplementary information

Reporting Summary
